# Risky drinkers and their physicians

**DOI:** 10.1186/1940-0640-10-S2-P11

**Published:** 2015-09-24

**Authors:** Hana Sovinova, Ladislav Csemy

**Affiliations:** 1 Monitoring and research center for tobacco and alcohol, National Institute of Public Health, Prague, Czech Republic; 2 National Institute of Mental Health, Klecany, Czech Republic

## Background

The study is focused on prevalence of risky and harmful drinking in Czech adults and on opinions of Czech GPs about the impact of alcohol on health of their patients. Self-assessment of doctors' effectiveness in reduction of patients' alcohol consumption was also addressed.

## Material and methods

The results presented are based on the National Survey on Tobacco Smoking and Alcohol Consumption (N = 1,802) [1,2] and on a survey of 294 Czech GPs carried out within the ODHIN project [3,4].

## Results

Risky or harmful alcohol consumption was found in one in five Czech adults. 34% of respondents reported their doctor asked them about alcohol consumption and 8.6% received advice to reduce or stop drinking. This advice was more common for respondents over 45 years of age. In contrast with this only 22 respondents (1.2%) felt they would need professional help[1,2]. About one third of the sample of Czech GPs considered None or very low drinking as very important for good health. Greater importance doctors attributed to other risk behaviors. The study identified lack of time, lack of professional training and lack of funding for preventive activities as barriers to broader implementation of alcohol consumption screening and brief intervention (SBI). Only 8.5% of GPs reported they were very effective in influencing drinking habits among their patients. 32% subjectively believe their efficiency in this area would be increased with adequate specialized training[3].

## Conclusions

Only eight to nine percent of PHC patients receive recommendation to reduce drinking. GPs feel insecure regarding SBI to reduce alcohol consumption in patients, which could be changed if adequate education is provided. To overcome major barriers of wider implementation of SBI in PHC joint efforts and common interest of GPs, health insurance agencies and the Ministry of Health are needed.
